# Immunohistological Evaluation of Peri‐Implant Soft Tissue Grafting Using a Porcine Dermal Matrix—A Human Histological Study

**DOI:** 10.1111/clr.70088

**Published:** 2026-01-30

**Authors:** F. R. S. Michallek, M. Sluka, V. C. Landwehr, K. Vach, L. Larsson, K. Nelson, S. Nahles, F. Kernen, G. Iglhaut, T. Fretwurst

**Affiliations:** ^1^ Department of Oral and Maxillofacial Surgery/Translational Implantology, Center for Dental Medicine, Medical Center, Faculty of Medicine University of Freiburg Freiburg Germany; ^2^ Institute of Medical Biometry and Statistics, Faculty of Medicine and Medical Center University of Freiburg Freiburg Germany; ^3^ Department of Periodontology, Institute of Odontology, Sahlgrenska Academy University of Gothenburg Gothenburg Sweden; ^4^ Department of Oral and Maxillofacial Surgery Charite—Universitätsmedizin Berlin, Corporate Member of Freie Universität Berlin, Humboldt‐Universität Zu Berlin, Berlin Institute of Health Berlin Germany; ^5^ Department of Oral Medicine, Infection, and Immunity Harvard School of Dental Medicine Boston Massachusetts USA

## Abstract

**Introduction:**

The aim was the immunohistological evaluation of a porcine dermal matrix (PDM) in comparison to a non‐augmented control group for peri‐implant tissue thickening.

**Materials and Methods:**

This human histological study involved the placement of PDM in the test group (20 patients) during implant placement, while the control group underwent implant placement without grafting (20 patients). Postoperative clinical evaluations were conducted, and biopsies were obtained after 3 months. Histomorphometric evaluation utilized H&E staining. Immunohistological analysis included CD31, PSR, TE‐7, CD3, CD20, CD138, and CD68.

**Results:**

A total of 40 patients (17 females, 23 males; 60.62 years) were included. Though more frequent gingival redness occurred during wound healing in the PDM, the groups showed no significant differences in epithelial thickness (*p* = 0.63), rete ridges (*p* = 0.53), papilla density (*p* = 0.626), papilla complexity (*p* = 0.053), vascularization (*p* = 0.052), connective tissue morphology (*p* = 0.127), collagen differentiation (*p* = 0.41), and CD3^+^ (*p* = 0.85) and CD138^+^ cells (*p* = 0.33). The PDM group showed significantly more CD68^+^ cells (*p* = 0.049) and CD20^+^ cells (*p* = 0.046), which correlates with a potentially distinct immune response caused by the PDM.

**Conclusion:**

The PDM used in this study exhibited no significant differences regarding epithelial changes, vascularization, tissue morphology, and collagen differentiation compared to a healthy control sample 3 months after grafting. The PDM group demonstrated a significantly higher frequency of CD68^+^ and CD20^+^ cells. The noticeable interindividual variation in immunoprofiles and clinical relevance of these findings should be investigated in future research.

**Abbreviations**
CD: cluster of differentiation
CTG: connective tissue graft
H&E: hematoxylin and eosin
IHC: immunohistochemistry
PCM: porcine collagen matrix
PDM: porcine dermal matrix
PLM: polarisation microscope
PSR: Picro‐Sirius‐Red
ROI: region of interest

## 1 Introduction

The quality and quantity of peri‐implant soft tissue is crucial for the long‐term stability and aesthetic outcome of implant‐supported restorations (Roccuzzo et al. 2023). A lack of peri‐implant soft tissue can lead to peri‐implant infections due to an insufficient mechanical and biological barrier protecting the peri‐implant area from bacterial invasion (Ikeda et al. 2002; Sorni‐Bröker et al. 2009). Moreover, greater gingival thickness helps prevent postoperative recessions, which are correlated with marginal bone loss, compromised oral hygiene (e.g., plaque accumulation, discomfort), and aesthetic drawbacks (Kao and Pasquinelli 2002; Romeo et al. 2004; Coli and Jemt 2021). When peri‐implant soft tissue is insufficient (< 2 mm), autologous free gingival grafts (FGG) or connective tissue grafts (CTG) can be used for soft tissue grafting. Although considered the gold standard, CTGs are associated with donor site morbidity and postoperative discomfort (Griffin et al. 2006; Cairo et al. 2019). To avoid complications associated with donor site harvesting, porcine collagen matrices (PCM) and, more recently, porcine dermal matrices (PDM) have been introduced in dentistry for use in gingival recession treatment and peri‐implant soft tissue grafting (Thoma et al. 2011; McGuire and Scheyer 2014). PCMs are produced from collagen‐rich tissues through extensive washing and enzymatic treatment, removing all non‐collagenous components and yielding purified collagen that is reconstituted into membranes (Ruszczak 2003). In contrast, PDMs are derived from porcine dermis without being cross‐linked artificially, where the native three‐dimensional architecture of collagen, elastin, and proteoglycans is preserved while cellular components are removed (Kirsner et al. 2015). Thus, PCMs consist of isolated collagen, whereas PDMs maintain the natural dermal scaffold structure. PDMs demonstrate favourable outcomes for tissue thickening in root coverage procedures; however, they do not outperform autologous connective tissue grafts in terms of mean and total root coverage (Iglhaut et al. 2024; Würflein et al. 2025). The PDM used in this study (“NovoMatrix”, *BioHorizons CAMLOG Biotechnologies GmbH, Basel, Switzerland*) has not yet undergone any clinical histological evaluation on humans.

To assess the influence of a PDM on wound healing, this study investigates parameters indicative of various aspects of soft tissue regeneration. CD31^+^ cells serve as markers of angiogenesis, which is crucial for tissue regeneration and nutrient supply (DeLisser et al. 1997). CD3^+^ (T‐lymphocytes), CD20^+^ (B‐lymphocytes), CD68^+^ (macrophages), and CD138^+^ (plasma cells) participate in inflammatory processes and are commonly observed during immune and foreign body reactions (Peña and Martin 2024). To evaluate the connective tissue architecture, collagen fibrils are analyzed using polarized light microscopy (Picrosirius Red staining, PSR), and the TE7 stain is examined to identify fibrosis or scar formation (Junqueira et al. 1978). The epithelial architecture is also an important indicator, as proper epithelial organization and maturation are essential for establishing an effective soft‐tissue barrier that protects the underlying implant from bacterial invasion and mechanical stress (Hosseini et al. 2020).

Over the past decade, few clinical studies performed on humans have investigated the biological response to porcine soft tissue substitutes (PCM or PDM) in peri‐implant soft tissue grafting focusing on at least one of the immunohistological parameters such as epithelial and connective tissue architecture, immune response, and/or angiogenesis (Parashis et al. 2014; Thoma et al. 2018; Puisys et al. 2019; Monteiro 2019; Manhal et al. 2021; Ashurko et al. 2022; Artzi et al. 2022, 2024). Sample sizes of the known existing literature immunohistologically analyzing porcine matrices for peri‐implant augmentation ranged from 4 to 30 patients, with most studies enrolling less than 15 patients. 50% of the available studies were descriptive, lacking comparable quantitative data (Parashis et al. 2014; Thoma et al. 2018; Monteiro 2019; Manhal et al. 2021). The available quantitative clinical studies are mostly performed on PCMs and generally reported minor or no significant differences between test and control groups (autologous grafts, preoperative mucosa) (Puisys et al. 2019; Ashurko et al. 2022; Artzi et al. 2022, 2024). A standardized histopathological approach looking at a broader spectrum of parameters for evaluating the healing response to PCM and especially PDM is lacking.

There is a need for a systematic understanding of how xenogeneic biomaterials for soft tissue grafting interact with the host tissues to optimize regenerative treatment strategies. Therefore, the aim of the present study was to perform an immunohistological evaluation of PDM in comparison to a non‐augmented control group for peri‐implant tissue thickening, focusing on epithelial and connective tissue architecture, immune response, and angiogenesis. The hypothesis of the present study was that there are no significant differences between the PDM and control groups 3 months after grafting.

## 2 Material and Methods

### 2.1 Study Design and Trial Registration

The study was designed as a human immunohistological study. The study protocol was approved by the ethics committee, Faculty of Medicine, University of Freiburg, Germany (No. 21‐1495) and is in accordance with the Declaration of Helsinki of 1975, revised in Fortaleza in 2013. All participants were informed and understood the objectives and the details of the study and signed a written informed consent document. It was registered at the Deutsche Register Klinischer Studien/German Clinical Trail Register (DRKS, DRKS00026732) and followed the STROBE guidelines (https://www.equator‐network.org/reporting‐guidelines/strobe/).

### 2.2 Patient Cohort

Patients were recruited from August 2021 to August 2023 at the Department of Oral‐ and Maxillofacial Surgery, Faculty of Medicine, University of Freiburg and Charité Berlin, Germany. Patients were included if an implant placement was indicated with or without a simultaneous soft tissue grafting. General health conditions contradicting dental implant therapy (e.g., uncontrolled diabetes mellitus), mucosal diseases (e.g., erosive lichen planus), and untreated endodontic were exclusion criteria. Patients with periodontal lesions with probing depth ≥ 4 mm adjacent to the planned implant position or general periodontitis/gingivitis were also excluded. Pregnant or lactating women, nicotine users, and patients participating in other studies were excluded in the present study. All patients reached the age of legal majority and presented with good oral hygiene (full mouth plaque score < 20%).

### 2.3 Intervention

Forty human study participants in total were assigned to either the test group (with PDM; 20 patients) or the control group (without PDM; 20 patients) based on their indication for soft tissue grafting. Indications for soft tissue grafting were defined as an objectifiable soft tissue deficit (< 2 mm in both vertical and horizontal dimensions) or a pre‐existing narrow or absent band of keratinized mucosa.

**FIGURE 1:**
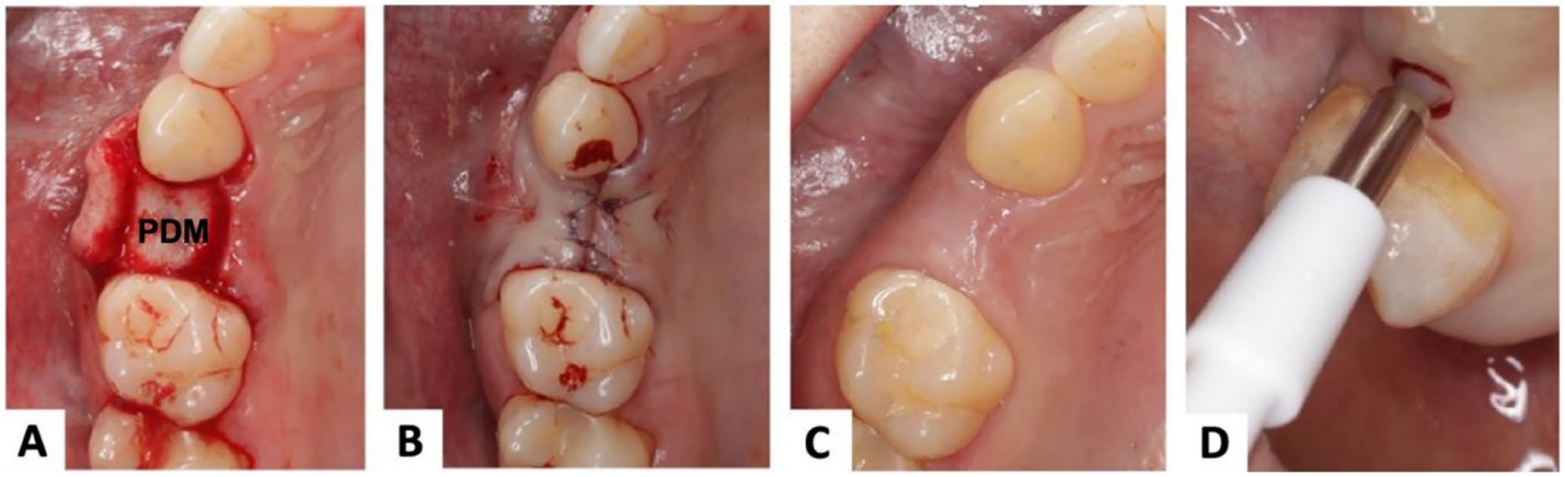
Soft tissue grafting with porcine dermal matrix (PDM) during implant placement. (A) Placement of the matrix over the dental implant for buccal and horizontal augmentation, (B) Suturing, (C) Clinical results after 3 months, (D) Transmucosal biopsy after 3 months (during implant exposure).

The surgical procedures required for sample collection were performed by surgeons from the Department of Oral and Maxillofacial Surgery, University Medical Center Freiburg and Charité Berlin. On the day of surgery, patients received 2000 mg of amoxicillin 1 h preoperatively for antibiotic prophylaxis. Following local infiltration anesthesia with Ultracain DS forte and mucosal disinfection, a horizontal incision was made along the alveolar ridge. The adjacent teeth were incised sulcularly, and a mucoperiosteal flap was elevated using a raspatory to assess the local bone morphology (Figure 1A). After exposure of the implant region and the buccal aspect of the alveolar bone, the implant was inserted according to the manufacturer's instructions. In the test group, the PDM was positioned over the placed implant to achieve buccal and horizontal soft tissue augmentation, whereas this step was skipped in the control group. The wound was then closed atraumatically and without tension over the graft (5–0 Monocryl, Ethicon Inc., Raritan, New Jersey, USA) (Figure 1B). No other hard or soft tissue augmentation was conducted. After a 3‐month healing period (Figure 1C), the implant was uncovered, and a gingival biopsy (2–3 mm in diameter) was obtained directly above the implant site using a punch (Figure 1D). For further processing, the biopsies were fixed in 3.5% neutral buffered Formalin.

Postoperative follow‐up was performed 1 day after surgery to assess complications such as infection, hematoma, postoperative pain, wound dehiscence, duration of analgesic use, and overall acceptance of the soft tissue grafting procedure. Sutures were removed 7–10 days postoperatively, and final wound control was carried out up to 30 days after surgery before the start of the prosthetic rehabilitation.

### 2.4 Sample Processing, Histochemistry

The biopsies were subsequently embedded in paraffin and cut into serial 3 μm thick sections for each stain on a rotary microtome (HistoCore Autocut, Leica, Wetzlar, Deutschland). H&E staining (Carl Roth GmbH + Co. KG, Karlsruhe, Germany) was performed for the histomorphometric evaluation. Collagen differentiation into collagen type I and III was detected using Picro‐Sirius‐Red (PSR) staining (Morphisto GmbH, Offenbach am Main, Germany) with a microscope (BZ‐X810, Keyence Deutschland GmbH, Neu‐Isenburg, Germany) equipped with a polarization filter (Daitron Co. LTD, Osaka, Japan).

### 2.5 Immunohistochemistry (IHC)

For IHC, sections were de‐waxed, and preprocessed via peroxidase block (3%, 10 min), antigen retrieval (80°C, 45 min; Citrate Buffer ZUC028‐100/500; Zytomed Systems GmbH, Berlin, Germany) and protein blocking (5 min; Blocking Solution ZytoChemPlus, Zytomed Systems GmbH, Berlin, Germany). The sections were incubated with the respective primary antibody for 45 min followed by incubation with a post block for 20 min. The secondary antibody kit used was conjugated to a horseradish peroxidase (30 min; ZytoChemPlus HRP Polymer anti‐Mouse, Zytomed Systems GmbH, Berlin, Germany). Positive cell visualization was accomplished using a DAB substrate (15 min; DAB Substrate Kit DAB530/057; Zytomed Systems GmbH, Berlin, Germany). Counterstaining was performed with hematoxylin solution according to Mayer.

The primary antibody panel included the following monoclonal, murine, anti‐human antibodies targeting the respective cell types:

- CD31/PECAM‐1: Endothelial cells
- TE‐7: Fibroblasts
- CD3: T‐lymphocytes
- CD20: B‐lymphocytes
- CD68/Macrosialin: Macrophages
- CD138/Syndecan‐1: Plasma cells

A detailed product list of the used antibodies and dilution rates is given in the Table A1.

### 2.6 Histomorphometry

The prepared serial sections were digitized using the Panoramic DESK II DW scanner (3D Histech/Sysmex, Budapest, Hungary) and analyzed with CaseViewer (3D Histech, Budapest, Hungary). Results were assessed by two trained investigators (FM, MS). Investigators were masked to patient clinical characteristics.

**FIGURE 2:**
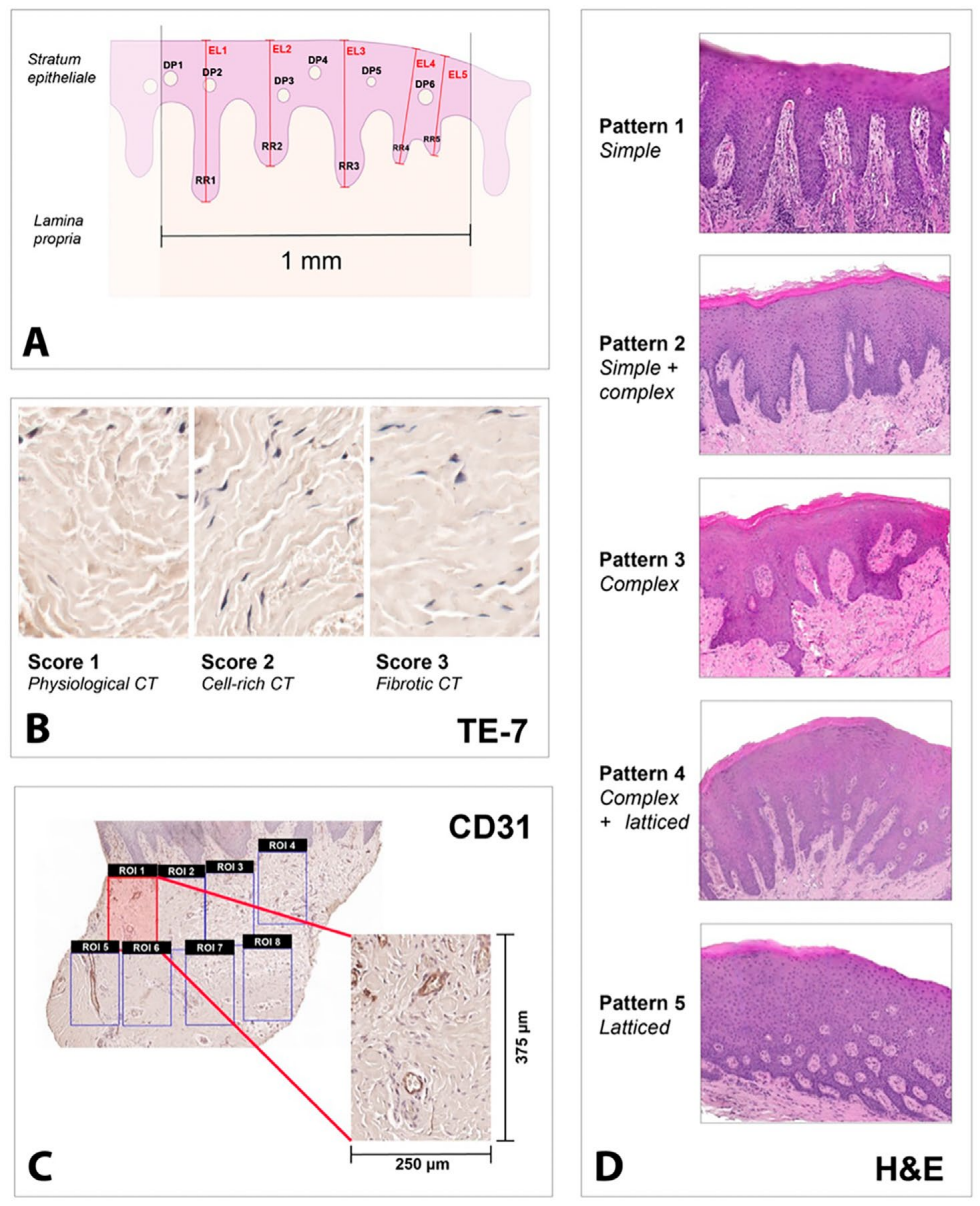
(A) Visualization of the assessed epithelial parameters. DP = dermal papilla, EL = epithelial length, RR = rete ridge. (B) Overview of the TE‐7‐stained connective tissue (CT) and different scores. (C) Exemplary CD31‐stained sample with selection of standardized ROIs within the CT. (D) Overview of the rete ridges patterns (H&E staining).

For epithelium discrimination, H&E sections were used. The assessed parameters were (1) epithelium thickness, (2) density of rete ridges (/1 mm), (3) density of dermal papillae (/1 mm) and (4) forms pattern of rete ridges (Figure 2A). The epithelium thickness (Str. epitheliale to Str. basale) was measured alongside the entire, continuous epithelium at the 5 thickest sites as described before (Thoma et al. 2018; Aragoneses et al. 2021). The density of rete ridges and dermal papillae was assessed within 1 mm of continuous epithelium, placed in the center of the horizontal length. Based on the assessment of rete ridges from Wu et al. (2013), an extended scoring system for rete ridges patterns was established, comprising the following patterns: Pattern 1: simple, Pattern 2: simple + complex, Pattern 3: complex, Pattern 4: complex + latticed, Pattern 5: latticed. The simple pattern (1) includes only individual straight rete ridges and does not contain any transversally cut dermal papillae. Pattern 2 consists mainly simple with components of initially complex rete ridges, which show a bifurcation at the bottom. The complex pattern (3) shows the highest degree of bifurcation and contains individual dermal papillae enclosed in the epithelium. Pattern 4 has less long complex rete ridges and an increased number of dermal papillae. The lattice form only consists of dermal papillae and short and isolated rete ridges (Figure 2D).

For the connective tissue analysis, the TE‐7 and PSR sections were used. Regions of interest (ROI; 250 × 375 μm) were placed to cover the whole sample. A scoring system was established and utilized for the TE‐7 sections where each ROI was assigned a score from 1 to 3. Score 1: Physiological connective tissue; loose arrangement of collagen fibers and scattered, spindle‐shaped cell nuclei (> 20 cells/0.01mm^2^). Score 2: Physiological connective tissue; richer in cell nuclei (> 20 cells/0.01mm^2^). Score 3: Fibrotic; parallel collagen fibers/dense connective tissue with less interfibrillar space and enlarged fibroblasts with rounded cell nuclei (Figure 2B).

In the PSR sections, collagen types were differentiated. Orange‐yellow fibers were assigned to collagen type I, green fibers to collagen type III. Crossways cut fibers or cell‐rich tissue could not be evaluated. The ROI assigned to the corresponding collagen types were counted and set as a percentage of the total ROI in a sample.

### 2.7 Immunohistochemistry Analysis

For IHC analysis, each sample was filled and covered with an average of 26 (min: 3; max: 78) randomly selected ROIs. The 250 μm × 375 μm rectangles were positioned across the entire sample in a non‐overlapping manner, ensuring that they included little or no epithelium and did not cover sample‐free or tissue‐empty areas. The ROIs were numbered in ascending order from the directly subepithelial region toward the deeper tissue layers. Images of the ROIs were captured and antibody‐positive cells (CD3, CD20, CD68, CD138) in the ROIs were counted using CaseViewer at 40× magnification. In the CD31 sections, CD31+ vessels were counted per ROI (Figure 2C).

### 2.8 Statistical Analysis

The sample size calculation was based on the results of a comparative study (Fretwurst et al. 2021). In the present study, a lower standard deviation of the measured parameters was anticipated due to greater homogeneity within the patient population. With 20 patients, a rate of 10% can be detected with a 95% confidence interval ranging from 0.01 to 0.0317. The calculation of group differences was based on the CD68 parameter results from Fretwurst et al. To detect a mean difference of 100 between the two groups with a power of 80%, 20 patients per group are required, assuming a two‐sided test with a significance level of 5%. Statistical analysis was performed using STATA 17.0 software (StataCorp LLC, Texas, USA). The significance level was set at 5%. For descriptive statistics, the median, mean, standard deviation, minimum, and maximum values were calculated. To visually present the results, box plots were created to illustrate the distribution of values in the control and PDM groups.

For statistical evaluation of CD31, CD3, CD20, CD68, CD138 expression, papilla density, and rete ridge density, the two‐sample Mann–Whitney U test was applied. Connective tissue morphology (TE7) and collagen type expression (PSR) were evaluated using a Pearson‐Chi‐Quadrat‐test.

## 3 Results

A total of 40 patients (23 males and 17 females; mean age 60.62 ± 13.46 years) were included in the present study. The mean patients age between the groups was comparable (PDM: 61.15 ± 11.52 years; control: 60.1 ± 15.44 years). Patient data, biopsy sites and placed implant systems are given in Table 1.

**TABLE 1:** Implant/patient data.

		All patients	CONTROL	PDM
		*N* = 40	*N* = 20	*N* = 20
Age (mean ± SD years)		60.62 (± 13.46)	60.1 (± 15.44)	61.15 (± 11.52)
Gender (male/female)		23/17 (57.5/42.5%)	10/10 (50/50%)	13/7 (65/35%)
Implant system		Camlog (67.5%) BioHorizons (10%) Straumann (7.5%) SIC (5%) Others (10%)	Camlog (70%) SIC (10%) Others (20%)	Camlog (65%) BioHorizons (20%) Straumann (10%) Others (5%)
Anterior (4–4)	Maxilla Mandibula	14 (35%)	6 (30%)	8 (40%)
		4 (10%)	2 (10%)	2 (10%)
Posterior (5–8)	Maxilla Mandibula	11 (27.5%)	9 (45%)	2 (10%)
		11 (27.5%)	3 (15%)	8 (40%)
All regions	Maxilla Mandibula	25 (62,5%)	15 (75%)	10 (50%)
		15 (37.5%)	5 (25%)	10 (50%)

*Note:* Overview of patient data (age, sex) and of the implants from which peri‐implant tissue samples were obtained (implant system, location: anterior/posterior, maxilla/mandible).

The procedure was uneventful for all patients. No loss of the PDM occurred. Every individual who was included in the study completed the follow‐up protocol and was eligible for analysis. The biopsy site (anterior/posterior; maxilla/mandibula) depended on the site of implant placement.

The results of the histomorphometry and IHC analysis are presented in Table 2.

**TABLE 2:** Histomorphometric and IHC analysis.

	Control, *N = 20*		PDM, *N = 20*		*p*
	Mean + SD	Range	Mean + SD	Range	
**Epithelium (H&E), *N* = 30**					
Thickness (μm)	517.8 ± 146.4	287.9–789.3	493.1 ± 135.3	236.7–661.2	0.630
Rete ridges (n/mm^2^)	6.9 ± 2.2	4–10	7.66 ± 3.43	3–16	0.530
Papillae (n/mm^2^)	4 ± 4.81	0–14	5.26 ± 6.39	0–23	0.626
**Vascularization (CD31), *N* = 40**					
Vessels (n/mm^2^)	70.8 ± 40.3	9.2–157.0	48.2 ± 31.3	15.8–149.3	0.052
**Collagen differentiation (PSR), *N* = 40**					
Collagen Type I (%)	30.72		34.75		0.410
Collagen Type III (%)	69.38		65.25		
**Connective tissue morphology (TE‐7), *N* = 40**					
Score 1: Physiologic (%)	38.86		28.64		0.127
Score 2: Physiologic/cell‐rich (%)	58.15		66.72		
Score 3: Fibrotic (%)	2.99		4.63		
**Immune Response** (positive cells/mm^*2*^), ***N* = 40**					
T‐Lymphocytes (CD3)	31.8 ± 84.3	0–280.3	16.0 ± 41.5	0–170.9	0.851
B‐Lymphocytes (CD20)	24.3 ± 71.1	0–247.1	13.3 ± 21.4	0–77.0	**0.046**
Macrophages (CD68)	5.2 ± 11.4	0–45.0	7.1 ± 9.6	0–27.3	**0.049**
Plasma cells (CD138)	19.4 ± 74.8	0–336.8	11.2 ± 18.4	0–73.6	0.333

*Note:* Overview of the Results regarding epithelium (H&E), vascularization (CD31), collagen differentiation (PSR), connective tissue morphology (TE‐7) and immune response (CD3, CD20, CD68, CD138) showing sample numbers, mean + standard deviation (SD), range and *p*‐value.

### 3.1 Epithelium

**FIGURE 3:**
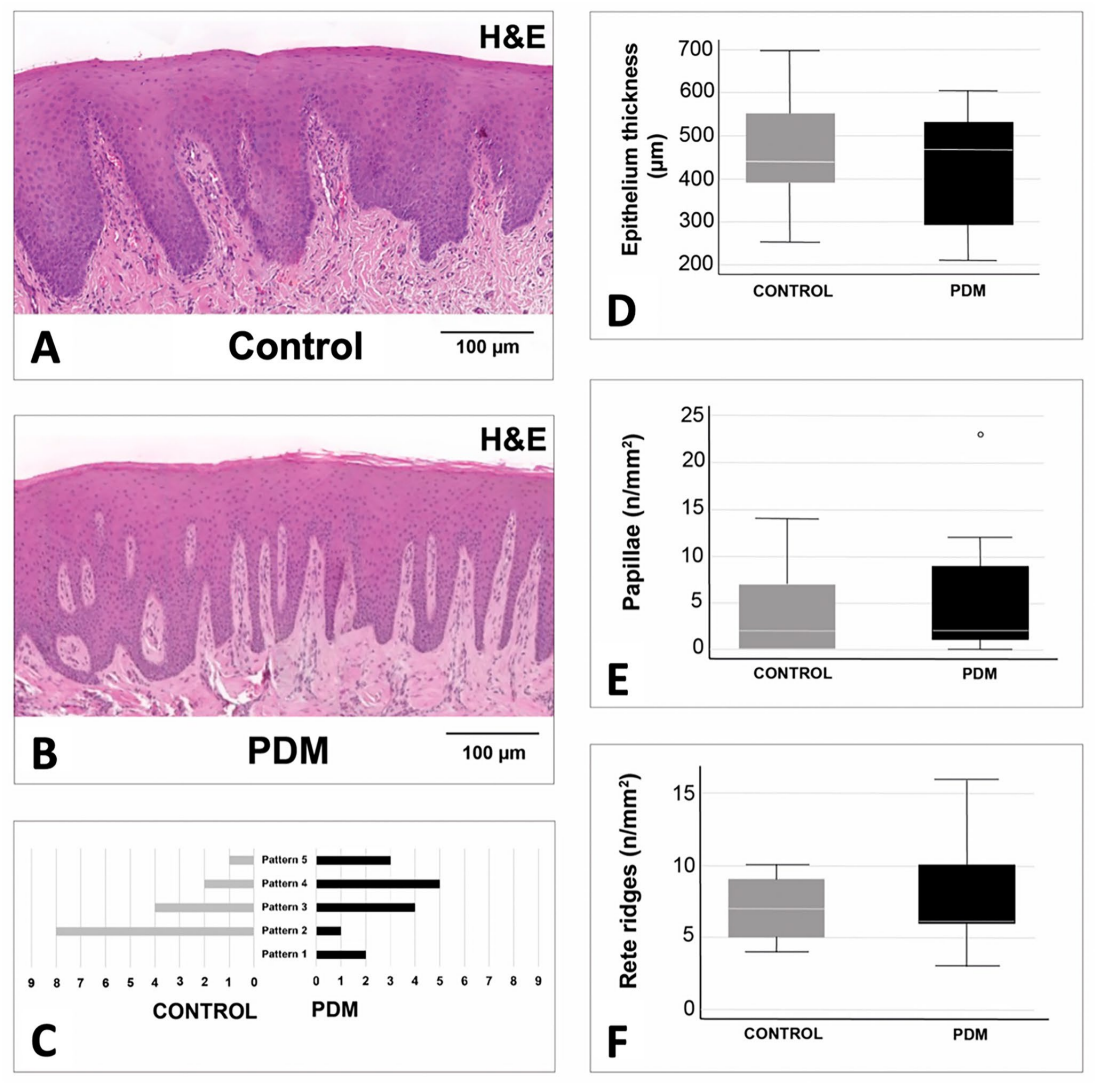
Histomorphometric comparison of the epithelium. (A) Epithelium of a representative control sample (H&E). (B) Epithelium of a representative PDM sample (H&E). (C) Distribution of the rete ridge patterns (control vs. PDM). (D) Boxplots visualizing the value distribution of the epithelium thickness (control vs. PDM). (E) Boxplots visualizing the value distribution of the dermal papillae density (control vs. PDM). Outlier marked with dot. (F) Boxplots visualizing the value distribution of the rete ridge density (control vs. PDM). H&E staining, magnification x40. Control = control group; PDM = porcine dermal matrix group.

30/40 biopsies (control *N* = 15, PDM *N* = 15) featured a closed continuous epithelium and, thus, were eligible for epithelium analysis. Histomorphometry of the epithelium demonstrated no significant differences between the two groups regarding the parameters investigated (Table 2 and Figure 3). The mean epithelial thickness in the control group was 517.81 (± 146.40) μm, whereas the mean thickness in the PDM group was 493.11 (± 135.34) μm (*p* = 0.630). The mean rete ridge density was 6.86 (± 2.16) in the control group and 7.66 (± 3.43) in the PDM group (*p* = 0.530). Control samples demonstrated a mean of 4 (± 4.81) dermal papillae per mm epithelium compared to 5.26 (± 6.39) in the PDM samples (*p* = 0.626).

The analysis of the rete ridges pattern revealed a spread across all pattern types in the PDM samples, while 8/15 samples in the control group featured pattern 2. However, no significant difference between the control and PDM groups was found regarding the rete papillae pattern distribution (*p* = 0.053).

### 3.2 Vascularization

**FIGURE 4:**
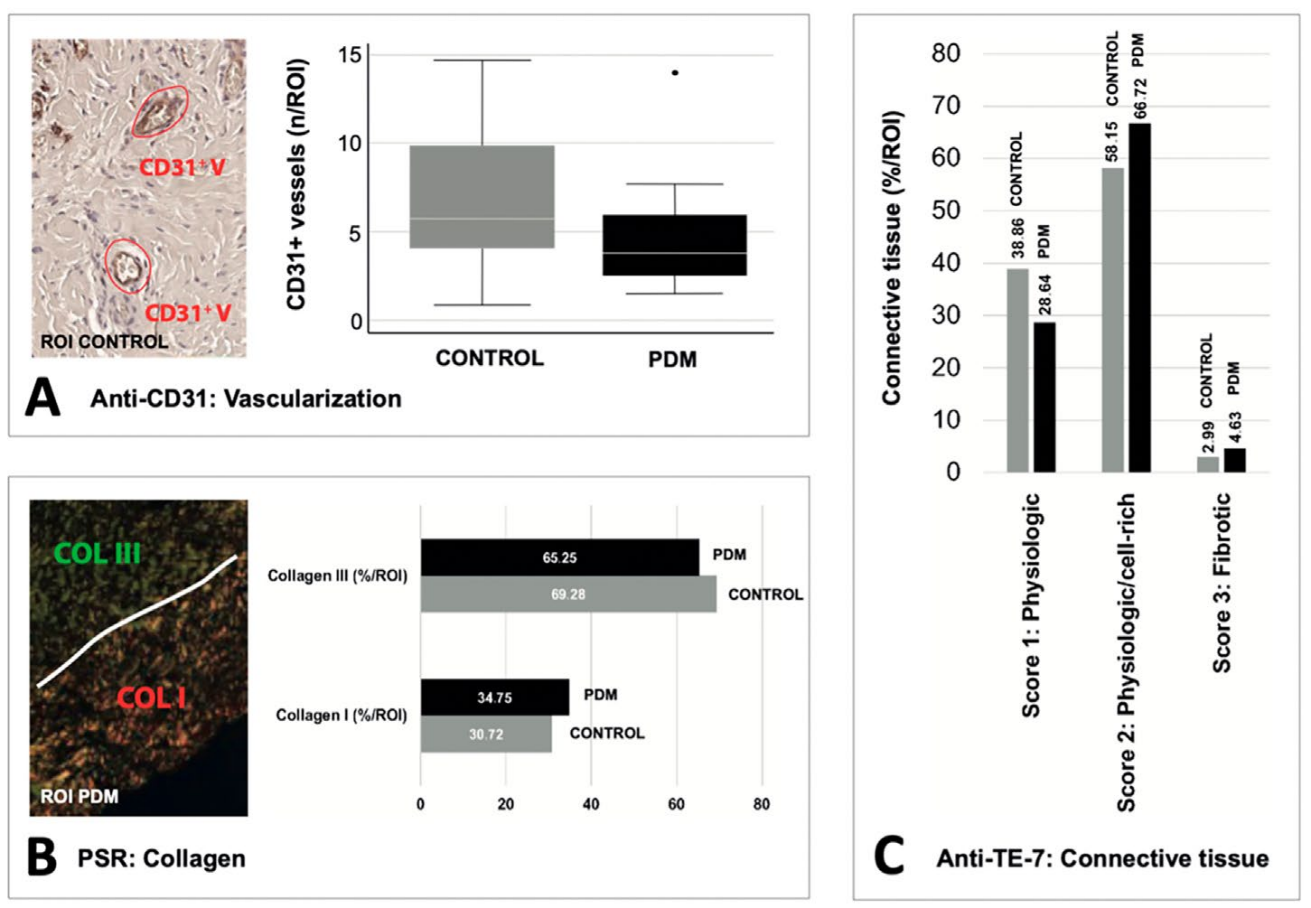
Immunohistological analysis of the connective tissue for control and PDM groups. (A) Vascularization (CD 31), (B) Collagen formation (picrosirius red with polarized microscopy, collagen type (CT) 1 and 3), (C) Fibroblast distribution (TE‐7, score 1–3). Magnification ×40. Control = control group, PDM = porcine dermal matrix group.

All 40 samples showed subepithelial connective tissue and could be included for analysis of the angiogenesis. There were no significant differences in vascularization between the PDM and control group. The control group demonstrated 6.64 ± 3.77 vessels/ROI, while the PDM group showed 4.52 ± 2.92 vessels/ROI (*p* = 0.052). Both groups demonstrated a broad range, with vessel density ranging from 0.86 to 14.72 vessels/total ROI in the control group and 1.48 to 14 vessels/ROI in the PDM group (Table 2 and Figure 4A).

### 3.3 Connective Tissue

All 40 samples showed subepithelial connective tissue and could be included for analysis of the connective tissue architecture. No remnants of the PDM material or foreign body giant cells were detected. No significant difference in the collagen type distribution was observed between the control group (collagen type I: 30.72%, collagen type III: 69.28%) and the PDM group (collagen type I: 34.75%, collagen type III: 62.25%, *p* = 0.41) (Figure 4B). No significant difference in fibroblast distribution was observed between the PDM and the control group (*p* = 0.127). Fibrotic tissue constituted 2.99% in the control group and 4.63% in the PDM group (Figure 4C).

### 3.4 Immune Response

All 40 samples showed subepithelial connective tissue and could be included for analysis of the presence of different immune cell types. For the antibodies CD3 and CD138, no significant differences were observed between the control group and the PDM group 3 months after healing (*p* = 0.85; *p* = 0.33). The control group demonstrated 31.8 ± 84.3 CD3^+^ cells/ROI, while the PDM group showed 16.0 ± 41.5 CD3^+^ cells/ROI (*p* = 0.85). For the CD138^+^ cells, the control group showed 19.4 ± 74.8 cells/ROI and the PDM group 11.2 ± 18.4 cells/ROI (*p* = 0.33).

**FIGURE 5:**
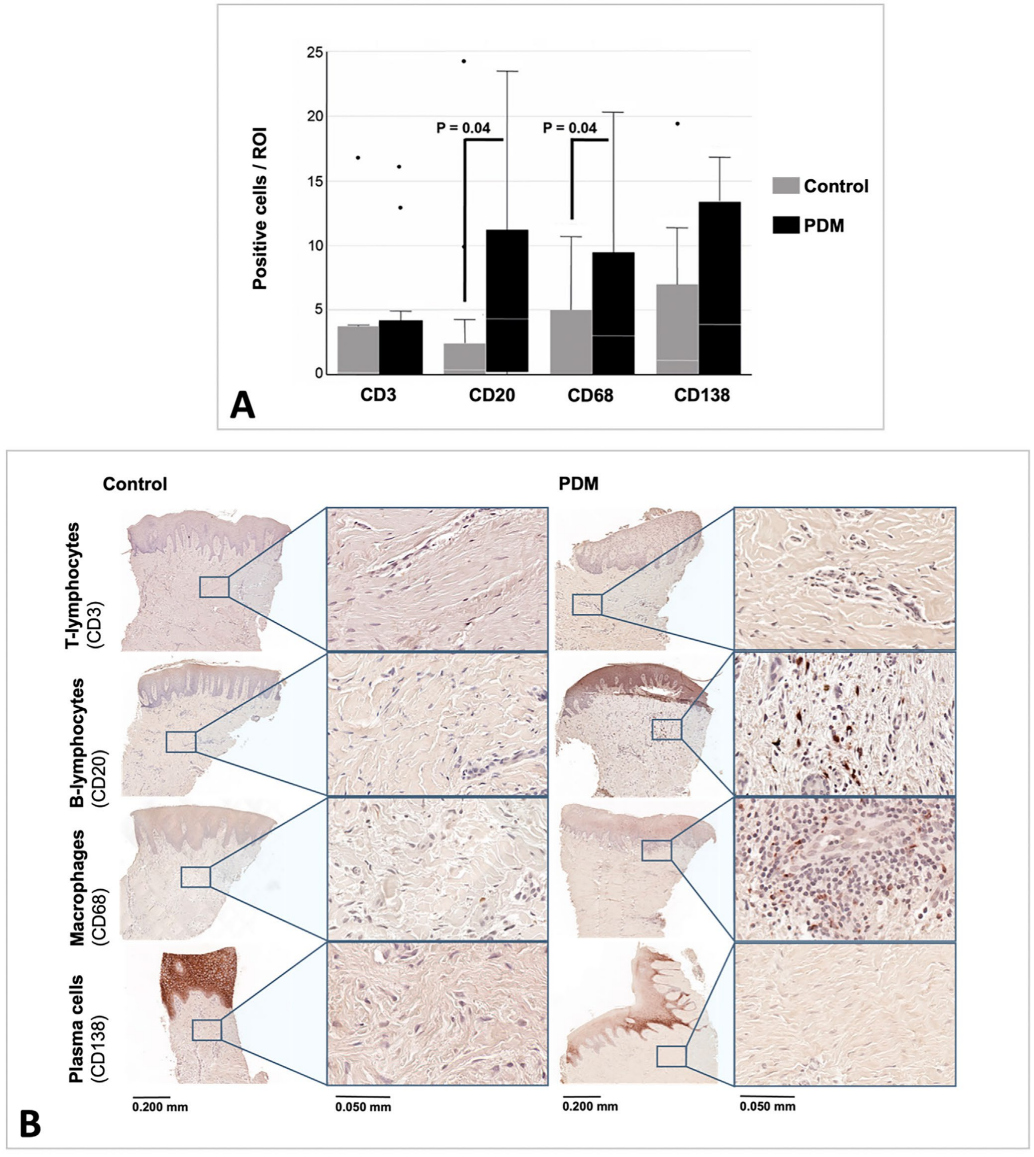
Immunohistological comparison of the immune response between control and PDM group. (A) Boxplots visualizing. (B) Representative samples. CD3: T‐lymphocytes, CD20: B‐lymphocytes, CD68: Macrophages, CD138: Plasma cells (Magnification: x5, x30; Scale: 0.200 mm; 0.050 mm). Control = control group, PDM = porcine dermal matrix group.

The PDM group exhibited a significantly higher frequency of CD68^+^ cells (Control: 5.2 ± 11.4 cells/ROI; PDM: 7.1 ± 9.6 cells/ROI; *p* = 0.049) and CD20^+^ cells (Control: 24.3 ± 71.1 cells/ROI; PDM: 11.2 ± 18.4 cells/ROI; *p* = 0.046) compared to the control group. Figure 5 visualizes the comparison of the immune response in the control group and group with PDM.

### 3.5 Interindividual Immune Cell Infiltrates

**FIGURE 6:**
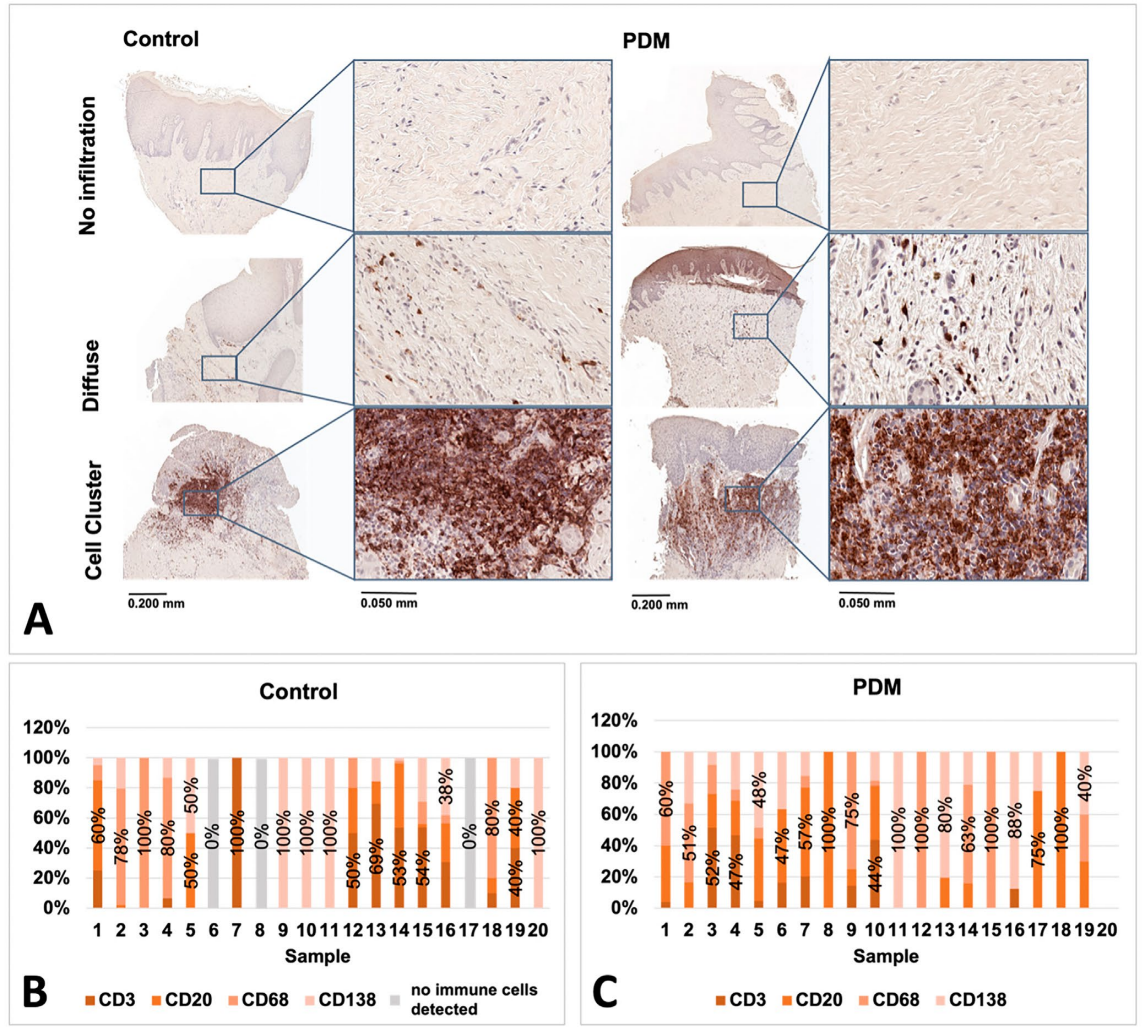
Interindividual immune cell infiltration of the PDM vs. control group. (A) Representative samples (no infiltration, diffuse infiltration, and cell clusters) of the PDM vs. control group. (B) Percentual distribution of different cell types in control group. (C) Percentual distribution of different cell types in the PDM group. CD3: T‐lymphocytes, CD20: B‐lymphocytes, CD68: Macrophages/Monocytes, CD138: Plasma cells (Magnification: x5, x30). Control = control group, PDM = porcine dermal matrix group.

Interindividual differences in immune cell infiltrates were observed (Figure 6B,C). In the control group, CD3+ and CD138+ cells were most frequent (27.5% each), followed by CD68+ (20%) and CD20+ (12.5%). No immune cells were detected in 15% of individuals (C6, C8, C17). In the PDM group, CD68+ cells were most common (30%), followed by CD20+ and CD138+ (25%) and CD3+ (15%). One individual (5%, PDM20) had no detectable immune cells.

As shown in Figure 6A immune cell infiltrated samples were significantly more frequently observed in the PDM group compared to the control group and samples without any infiltration were significantly more frequent in the control group (*p* = 0.021). Immune cell clusters were observed in both groups (*p* > 0.05).

## 4 Discussion

The present study aimed to compare the immunohistological characteristics of soft tissue healing in patients receiving dental implants with and without the application of a porcine dermal matrix (PDM) as a soft tissue grafting material. After a 3‐month healing period, there were no significant differences between the two groups in terms of epithelial architecture, angiogenesis, and collagen formation. However, the PDM group exhibited a significantly higher frequency of CD68^+^‐cells and CD20^+^‐cells, suggesting a potentially distinct immune response in these patients after 3 months. Interindividual differences in immune cell infiltrates were observed in both groups. Immune cell infiltration was significantly more frequent in the PDM group.

### 4.1 Epithelial Architecture and Angiogenesis

No significant differences for epithelial thickness, rete ridge density, and dermal papillae density were observed between the control and PDM group. This suggests that the PDM used in the present study did not have an influence on the organization or morphology of the epithelium. These results are consistent with previous studies that also reported no significant effects of soft tissue grafting materials on epithelial healing (Vignoletti et al. 2015; Thoma et al. 2018; Artzi et al. 2022). Angiogenesis, as evaluated by CD31 staining, demonstrated no significant difference between the groups. The lack of a significant difference in vascularization between the two groups might be attributed to the fact that the matrix was already integrated by the 3‐month time point, allowing for similar angiogenesis in both groups. Comparable studies demonstrated no significant differences in vessel density or size for CD31 staining between PDM and a control area, as well as for PCM as a grafting material (Puisys et al. 2019; Artzi et al. 2022), whereas Artzi et al. (2024) reported an increased number of CD31‐positive cells after soft tissue grafting with a PCM after 3 months (Artzi et al. 2024).

### 4.2 Connective Tissue Architecture

No remnants of the PDM or foreign body giant cells were detected in the biopsies, indicating complete integration/remodeling or resorption of the matrix. Since latest studies demonstrated persistent soft tissue volume around teeth following recession coverage after one and two year with the same PDM used in this study, this could indicate that the matrix is integrated/remodeled rather than resorbed (Würflein et al. 2025). In particular, as no fibrosis or scar tissue formation was observed in the present study indicated by the TE‐7 results. It seems that the PDM material does not induce excessive fibrosis, which is consistent with the reported biocompatibility of porcine‐derived matrices (Azab and Youssef 2021; Almeida et al. 2024). This is in accordance with studies performing tissue grafting with PCMs in animal models, which also could not detect the applied soft tissue substitutes after 3 months of follow‐up (Jung et al. 2011; Caballé‐Serrano et al. 2019).

Both groups exhibited similar distributions of collagen types I and III, indicating no substantial effect of the PDM material on collagen deposition. Similarly, two quantitative studies in animal models could not detect any differences in Collagen I expression between control and PDM groups or PCM groups respectively (Schmitt et al. 2019; Lee et al. 2023). A comparable clinical study analyzing fibroblasts 3 months after grafting with a PCM instead of a PDM found no significant difference in fibroblast numbers compared to the control group (Artzi et al. 2024). Artzi et al. used preoperative sites as the control group whereas this study looked at postoperative implant sites with no augmentation which could interfere with the amount of scarring seen at the different sites.

### 4.3 Immune Response

The PDM group exhibited a significantly higher frequency of CD68^+^‐cells and CD20^+^‐cells compared to the control group. This increased macrophage and lymphocyte immune response is likely not due to the procedure (implant placement) itself, as it did not occur in the control group. Macrophages and lymphocytes play a critical role in tissue remodeling and inflammatory processes. The significantly increased presence of CD20^+^ and CD68^+^ cells, along with the notably higher number of samples in the PDM group showing diffuse immune cell infiltration, indicates a tendency toward a heightened immune response associated with a prolonged wound healing phase due to the PDM (Wynn and Vannella 2016; Peña and Martin 2024). This may be caused by residual molecular xenogeneic components remaining in the matrix after processing, which could potentially trigger an immune response. Such stimulation might have led to the upregulation of immune cells and the diffuse infiltration observed in almost all PDM samples. However, due to the borderline statistical significance of the increased CD20^+^ and CD68^+^ cell counts, these findings should be interpreted with caution. This is especially true since, despite more gingival redness in the PDM group, no clinically relevant effects were observed after the 3‐month healing period, and there were no impacts on other parameters. The increased number of diffusely infiltrated samples in the PDM group most likely represents an elevated baseline immune cell activity, correlating with a physiological response following a surgical procedure such as soft tissue augmentation. In addition, no significant differences were observed in the frequency of CD3^+^ T‐lymphocytes or CD138^+^ plasma cells between the two groups.

### 4.4 Interindividual Differences in Immune Cell Infiltrates

One interesting finding in this study was the observation of interindividual differences in immune cell infiltrates. The composition and abundance of immune cell types varied considerably in individual samples, with some individuals showing predominantly lymphocytic infiltrates, while others contained higher proportions of macrophages or plasma cells. In a few individuals, no immune cells were detected at all. These findings highlight the heterogeneous nature of the local immune response, which may depend on patient‐specific factors such as tissue characteristics, age, systemic health, or individual variability in immune activation (Brodin and Davis 2017; Kaczorowski et al. 2017). Localized clusters of immune cells were observed at similar frequencies in both groups, suggesting that these clusters are likely unrelated to PDM and may instead reflect other local, individual inflammatory processes.

### 4.5 Limitations and Outlook

Samples were evaluated after a 3‐month healing period which did not capture long‐term effects. A longer follow‐up period could provide valuable insights on potential complications associated with the PDM. Even though all assessors were calibrated by an experienced histologist prior to the evaluation, including a greater number of independent assessors would further strengthen the validity of the results. A larger sample size could help identify smaller but clinically meaningful differences. Including relevant clinical parameters would also improve the clinical applicability and relevance of the findings. Although the patients' age and the anatomical region of implant placement were documented, the sample size is too small to draw definite statistical conclusions regarding their relevance. Differences in age and implant region can be observed between the two groups, which may influence the immunological response. However, previous research suggests that age has no significant effect on the outcome of implant treatments (Boboeva et al. 2021). Other possible confounders, such as individual immune variability (as presented in the study), further systemic health factors, nutrition, or oral microbiome composition, were not accounted for and may have influenced the results. This should be included in future studies in the field.

While the clinical impact of the observations made in this study remains unclear, they may reflect the dynamic and individual‐specific nature of tissue healing, emphasizing the need for personalized approaches in regenerative medicine (Halstenbach et al. 2024). Especially since immunological interindividual differences are observed in the oral environment (Fretwurst et al. 2021, 2022; Williams et al. 2021; Halstenbach et al. 2023). In future research, it would be interesting to investigate how the immune response differs in individuals with immunologically altered medical histories and how such pre‐existing conditions influence wound healing on both immunohistological and clinical levels. Furthermore, proteomic analyses could provide insights into differences in pathways involved in inflammatory signaling, connective tissue remodeling, or angiogenesis. It should also be considered whether modifications in the processing of the matrix could improve the immunohistological response of peri‐implant tissues, a prospect that may have relevant implications for material development and industrial application.

## 5 Conclusion

In conclusion, the application of a PDM as a soft tissue grafting material during dental implant placement did not lead to significant differences in epithelial healing, collagen formation, or vascularization. However, the PDM group demonstrated a higher frequency of macrophages and B lymphocytes, which may suggest a more pronounced immune response in comparison to the control group. Despite these immunological differences, there were no signs of fibrosis or scar tissue formation, and the overall healing process appeared uneventful. The interindividual variability observed in immune cell infiltrates underscores the complex and personalized nature of tissue healing. The clinical relevance of these findings has yet to be further investigated. As it stands, no clinical complications in wound healing were observed in association with the PDM in this study. Therefore, despite the tendency toward a heightened immune response, the PDM may be considered a viable alternative to the current gold standard, the autologous connective tissue graft (CTG) by eliminating the risk of donor site morbidity.

## Author Contributions

**F. R. S. Michallek:** methodology, formal analysis, writing – original draft, visualization, funding acquisition. **M. Sluka:** methodology, formal analysis, writing – original draft, visualization. **V. C. Landwehr:** conceptualization, methodology, formal analysis, investigation, writing – review and editing, funding aquisition. **K. Vach:** formal analysis, writing – review and editing, visualization. **L. Larsson:** formal analysis, writing – review and editing. **K. Nelson:** conceptualization, methodology, investigation, resources, writing – review and editing, supervision. **S. Nahles:** investigation, resources, writing – review and editing. **F. R. Kernen:** investigation, writing – review and editing. **G. Iglhaut:** conceptualization, investigation, writing – review and editing, supervision, funding aquisition. **T. Fretwurst:** conceptualization, methodology, formal analysis, resources, writing – original draft, supervision, funding acquisition.

## Funding

The study was supported by a research grant from the Oral Reconstruction Foundation (ORF Grant 42108). F.R.S.M. was supported by a Doctoral‐Scholarship of the Hanns‐Seidel‐Stiftung (German Federal Ministry of Education and Research (BMBF)).

## Ethics Statement

The study was approved by the ethics committee of the Faculty of Medicine, University of Freiburg, Germany, No 21‐1495. Before enrollment, informed consent was obtained from all individual participants included in the study.

## Consent

The authors have nothing to report.

## Conflicts of Interest

The authors declare no conflicts of interest.

## Supporting information


**Data S1:** Supporting information.

## Data Availability

All data generated or analyzed during this study are included in this published article and its Supporting information files.

## Appendix A

**TABLE A1:** Primary antibodies used for IHC.

Antibody	Product	Dilution
CD31	DAKO M0823 monoclonal, mouse anti‐human, DakoCytomation A/S, Glostrup, Dänemark	1:70
TE‐7	Merck CBL271, monoclonal, mouse anti‐human, Merck KGaA, Darmstadt, Germany	1:2000
CD3	DAKO M7254, monoclonal, mouse anti‐human, DakoCytomation A/S, Glostrup, Dänemark	1:50
CD20	DAKO M0755, monoclonal, mouse anti‐human, DakoCytomation A/S, Glostrup, Dänemark	1:600
CD68	DAKO M0814 clone PG‐M1, monoclonal, mouse anti‐human, DakoCytomation A/S, Glostrup, Dänemark	1:200
CD138	DAKO M7228 clone MI15, monoclonal, mouse anti‐human, DakoCytomation A/S, Glostrup, Dänemark	1:70
